# Association of Tear Osmolarity With Signs and Symptoms of Dry Eye Disease in the Dry Eye Assessment and Management (DREAM) Study

**DOI:** 10.1167/iovs.64.1.5

**Published:** 2023-01-10

**Authors:** Jack V. Greiner, Gui-shuang Ying, Maxwell Pistilli, Maureen G. Maguire, Penny A. Asbell, for the Dry Eye Assessment and Management (DREAM) Study Research Group

**Affiliations:** 1 Schepens Eye Research Institute of Massachusetts Eye & Ear Infirmary, Department of Ophthalmology, Harvard Medical School, Boston, Massachusetts, United States; 2 Department of Ophthalmology, Perelman School of Medicine, University of Pennsylvania, Philadelphia, Pennsylvania, United States; 3 Department of Ophthalmology, Hamilton Eye Institute Health Science Center, The University of Tennessee, Memphis, Tennessee, United States

**Keywords:** DREAM, Dry Eye Assessment and Management (DREAM) study, dry eye disease, OSDI, Schirmer's test, tear film break-up time, tear osmolarity, vital staining

## Abstract

**Purpose:**

To determine the relationships of (1) tear osmolarity (TO) levels with the severity of signs and symptoms of dry eye disease (DED) and (2) changes in TO with changes in signs and symptoms.

**Methods:**

Patients (N = 405) with moderate to severe DED in the Dry Eye Assessment and Management (DREAM) Study were evaluated at baseline and at six and 12 months. Associations of TO with signs and symptoms were evaluated using Pearson correlation coefficient (*r*) and regression models.

**Results:**

The mean (standard deviation [SD]) TO was 303 (16) mOsm/L at baseline and 303 (18) mOsm/L at both six and 12 months. TO was higher in older patients (306 mOsm/L for ≥70 years vs. 300 mOsm/L for <50 years; *P* = 0.01) and those with Sjögren's disease (311 vs. 302 mOsm/L; *P* < 0.0001). TO did not differ between patients randomized to placebo and omega-3 fatty acid supplementation. TO was weakly correlated with conjunctival (*r* = 0.18; *P* < 0.001) and corneal staining scores (*r* = 0.17; *P* < 0.001), tear film break-up time (*r* = 0.06; *P* = 0.03), and Schirmer test score (*r* = −0.07; *P* = 0.02) but not with Ocular Surface Disease Index scores (*r* = 0.03; *P* = 0.40). Changes in signs and were not significantly correlated with change in TO at six or 12 months.

**Conclusions:**

Within DREAM, TO was weakly correlated with DED signs, explaining <5% variability in signs. Changes in tear osmolarity were not associated with changes in signs and symptoms of DED, indicating that the association may not be causal.

Since 1970, when Mishima and colleagues found increased tear osmolarity (hyperosmolarity) in patients with dry eye compared to normal subjects, this relationship has been well documented.^1^ Tear hyperosmolarity has been defined as one of the two core mechanisms of dry eye disease (DED) regardless of cause.^2^ Because tear film hyperosmolarity appears to be pathognomonic for DED, some have considered it the single best marker for DED.^3^ Hyperosmolarity may be responsible for ocular surface symptoms associated with DED,^2^^,^^4^^–^^6^ although cautious interpretation must be practiced ^7^ because a non-DED diagnosis may be present in patients with dry eye symptoms having normal tear osmolarity.^8^ Hyperosmolarity has been described as perpetuating a cycle of ocular surface inflammation and tear film instability.^9^ The determination of tear osmolarity is dependent on the interaction of tear production, evaporation, and drainage, all of which contribute to its measurement. Because hyperosmolarity is recognized as a key pathophysiological mechanism of DED,^2^^,^^10^ decreasing osmolarity may suggest improving DED.

Although Bron and Willshire^1^ present literature on a range of normal values, osmolarity ≤308 mOsm/L is considered normal.^11^ To provide some context for DED, an osmolarity of 316 mOsm/L can be regarded as the threshold between mild and moderate-severe dry eye.^12^^,^^13^ A difference of >8 mOsm/L between eyes is proposed to be suggestive of DED.^9^ Previous reports on correlation of tear osmolarity with signs and symptoms of DED have not been consistent.^5^^,^^14^^–^^19^ This may be due to the high degree of variability in DED patients along with the difficulties encountered with attempts to control variable conditions.^14^ Moreover, even though it is generally accepted that tear osmolarity is elevated in patients with DED, some studies report elevated tear osmolarity in subjects categorized as normal compared to subjects with DED.^20^^,^^21^ In addition, the coefficient of repeatability of osmolarity has been reported to be 33 mOsms/L, and any change smaller than this could be attributed to measurement noise.^16^ Although variability in osmolarity measurement may occur with poor operator training or device inaccuracy,^15^ good accuracy of office osmolarity measurements has been demonstrated in both in vitro solutions^22^^,^^23^ and non-DED patients.^24^ As discussed previously, there is a significant overlap in osmolarity measurements between DED patients and “normal” subjects.^23^^,^^25^ Moreover, patients with DED have day-to-day variability that is greater than in normal subjects^5^ and additionally possible diurnal variations.^26^^–^^29^ These prior studies included relatively small numbers of subjects. A large population of subjects would be expected to provide more definitive results on the associations between osmolarity and DED symptoms and signs.

The multi-center Dry Eye Assessment and Management (DREAM) study of 535 DED subjects evaluated the effects of high levels of omega-3 fatty acid supplementation on signs and symptoms in patients with moderate to severe DED with the hypothesis that dietary supplementation would improve dry eye symptoms and signs.^30^ Results of this randomized double-masked trial showed no difference in symptoms after one year of treatment between the active and placebo group and little change in signs in either group. However, this well-characterized DED cohort with standard examinations over one year allowed for evaluation of tear osmolarity in DED subjects with moderate to severe DED and to assess the associations of tear osmolarity with signs and symptoms and their changes over time.

## Methods

### Subjects

The DREAM Study was a prospective, randomized, double-masked, clinical trial designed to determine efficacy and safety of oral omega-3 fatty acid supplements for the treatment of DED.^30^ The study enrolled 535 participants from 27 clinical centers in 17 states of the United States who were randomly assigned to either an active omega-3 fatty acid supplement group or a placebo group (Supplementary Material). Approval was obtained from Institutional Review Board /Ethics Committees and adhered to the tenets of the Declaration of Helsinki. All centers were compliant with the Health Insurance Portability and Accountability Act, and written informed consent was obtained. All enrolled participants had at least one eye in compliance with the DREAM Study criteria for dry eye. Tear osmolarity measurements were performed at 19 clinical sites that had the needed equipment, which allowed analysis of data from 405 subjects over one year of evaluation.

#### Inclusion Criteria

Subject inclusion criteria included age ≥18 years; dry eye–related ocular symptoms for at least six months before the screening visit and the use or desire to use artificial tears on average twice daily for two weeks before the screening visit. Participants had to qualify with symptoms of dry eye based on the Ocular Surface Disease Index (OSDI) score (ranging from 0 1o 100)^31^ of at least 25 (≥25 to ≤80) at the screening visit and at least 21 (≥21 to ≤80) at the eligibility confirmation visit. Additionally, participants had to demonstrate the presence of at least two of the four following signs in the same eye at the screening visit and eligibility confirmation visit: (1) tear film break-up time (TBUT) ≤7 seconds; (2) corneal sodium fluorescein staining score of ≥4 of a possible score of 15 per eye; (3) conjunctival lissamine green staining score ≥1 of a possible score of 6 per eye; (4) anesthetized Schirmer's test ≥1 to ≤7 mm/5 min. The study design included patient symptom assessments and clinical measurements at baseline and six- and 12-month visits.

#### Exclusion Criteria

Participants were excluded if they had worn contact lenses within 30 days before the study, had history of refractive surgery or any recent ocular surgery, ocular infection, or contraindications to high-dose omega-3 supplementation such as anticoagulant therapy. Patients regularly using treatments for DED, including omega-3 fatty acid oral supplements (eicosapentaenoic acid and docosahexaenoic acid <1200 mg by mouth daily), systemic medications associated with ocular dryness, systemic corticosteroids, or other immunosuppressive agents were permitted to continue these treatments if committed to using them for 12 months. Patients were excluded if they used <90% of run-in supplements (placebo soft gel capsules) on the days between the screening and randomization visits.

### Treatments

Patients were randomly assigned in a 2:1 ratio to groups receiving either active omega-3 supplement or placebo supplements for the 12-month study period. Both the active and placebo supplement groups required the daily ingestion of five soft gel capsules. Each active supplement capsule contained 400 mg of eicosapentaenoic acid and 200 mg of docosahexaenoic acid, totaling 2000 mg of omega-3 fatty acids. The placebo supplement each contained 1000 mg of refined olive oil, composed for 68% oleic acid, 13% palmitic acid, and 11% linolenic acid totaling a daily dose of 5000 mg of olive oil. This was equivalent to approximately 1 teaspoon of olive oil. Capsules were manufactured by the Access Business Group (Ada, MI, USA).

### Osmolarity Measurements

At the 19 clinical centers that had TearLab Osmolarity System (OcuSense Inc., San Diego, CA, USA), osmolarity measurement was taken at baseline and the six- and 12-month visits. At each center, both the clinician and technician completed a certification program that included review of the study protocol and instructional slides, and a written knowledge assessment of the tear film osmometer. Osmometer measurements were performed using an electrode to measure electrical impedance technology on 50 nL tear fluid samples in a TearLab Osmolarity System (OcuSense Inc.) operated according to the manufacturer's instructions. With the subject looking in primary gaze, the osmolarity test card tip was dipped into the tear film meniscus in the central portion of the temporal third of the lower eyelid. The osmometer was calibrated before each test using the control solution provided by TearLab. Patients were asked not to instill eye drops within two hours before any study visit examination. Before any diagnostic examination or instillation of any eye drops, tear osmolarity was measured once in each eye using disposable test cards provided by the manufacturer's osmometer system. Tear osmolarity was defined as normal if ≤308 mOsm/L and abnormal if >308 mOsm/L or if the measurement between the two eyes was >8 mOsm/L, based on the TFOS DEWS report.^9^

#### Vital Staining

Staining of the corneal and conjunctival epithelia of each eye was evaluated using the standard fluorescein strip method.^32^ A standardized post-instillation period for each of the vital stains began with sodium fluorescein stain to evaluate the corneal epithelium and followed with lissamine green stain to evaluate the conjunctival epithelium, according to DREAM study guidelines.^30^ Grades for all staining were based on methods previously described^11^ with a scale ranging from 0 (none) to 3 (severe) in each of five zones of the cornea (central, and equally divided inferior, superior, temporal, and nasal quadrants) in each eye^11^ with total score ranging from 0 to 15. Conjunctival staining grades used the same scale in each of six zones grading (far temporal, superotemporal, inferotemporal, superonasal, inferonasal, and far nasal) with a total score ranging from 0 to 18.^11^

#### Schirmer Test

A type I Schirmer Test was performed after instillation of one eyedrop of 0.5% proparacaine ophthalmic solution in all subjects. Filter paper Schirmer strips were standardized among all sites. After slight eversion of the lower eyelid the filter paper strip was placed gently on the distal portion of the tarsal conjunctiva of the lower lid. The lid was returned to the anatomical position with the remaining portion of the Schirmer strip extending over the lid margin outward. This procedure was repeated on the contralateral eye and the Schirmer Strip was positioned at the junction of the middle and temporal third of both eyes. Eyes were then gently closed. After five minutes, eyes were opened, the Schirmer's strip was removed, and measurement of the portion of the strip wetted by the tears was recorded in millimeters according to the manufacturer's instructions.

#### TBUT by Biomicroscopy

After fluorescein instillation, subjects were asked to blink naturally to spread the fluorescein over the surface of the cornea. Using the cobalt blue filter and a wide beam on the biomicroscope the cornea was examined to establish that the fluorescein was spread evenly. Once the fluorescein between blinks appeared evenly spread, the subject was asked to open the eyes, maintain primary gaze, and not to blink. The interval between opening the eye and the appearance of the first dark or black spot or region over the cornea was recorded in seconds for Tearfilm Break-up Time. After determination of the TBUT, the same procedure was performed on the contralateral eye.

#### NonInvasive Break-Up Time

The tear film noninvasive break up time was measured using the Oculus Keratography Topography unit (Oculus, Inc., Arlington, WA, USA). Noninvasive break-up time measurements were made in addition to the bulbar conjunctival redness, and tear film meniscus height in each eye. All measurements were made using the manufacturer's instructions at 13 of the 27 clinical centers that had an Oculus Keratography Topography unit.

### Statistical Analysis

Continuous measures were summarized using as mean ± standard deviation (SD). Comparisons between treatment and placebo groups for eye-specific tear osmolarity and comparisons of DED severity scores of symptoms and signs among osmolarity groups were performed using generalized linear regression models with application of generalized estimating equations to account for the correlation between measures for eyes of the same patient. For eye-level analysis of association between osmolarity and signs, osmolarity groups were defined by eye-level osmolarity (≤308, >308 to ≤316, >316 mOsm/L). For person-level analysis of osmolarity with dry eye symptoms and signs, person-level osmolarity groups were based on osmolarity abnormality (defined osmolarity >308 mOsm/L or intereye difference >8 mOsm/L) and the maximum tear osmolarity from two eyes of a patient (≤308, >308 to ≤316, >316 mOsm/L). For these evaluations of associations between osmolarity and DED signs and symptoms, we performed the statistical analysis for the combined data from all three time points (baseline, six months, and 12 months) to increase the statistical power. In these analyses, time was modelled as a categorical variable, and the correlation from repeated measures at three different time points were accounted for by using the generalized estimating equations. Separate analysis for baseline, six months, and 12 months was also performed to check the consistency of the findings over time. The correlation between osmolarity with DED signs and symptoms were summarized using Pearson correlation coefficient (*r*), and the corresponding *P* value was calculated from linear regression models with generalized estimating equations that account for correlation between measures for eyes of the same patient and the correlation from repeated measures over time. All statistical analyses were performed in SAS v9.4 (SAS Institute Inc., Cary, NC, USA), and two-sided *P* ≤ 0.05 was considered to be statistically significant.

## Results

The study included a total of 405 patients from 19 clinical centers with a TearLab Osmolarity System for measuring tear osmolarity. The mean age at enrollment was 58 years (SD = 13), 81% were female, 72% were Caucasian, 10% had Sjögren's disease. The mean (SD) baseline OSDI score was 44 (SD = 14). Patient and ocular characteristics at baseline are presented in Table 1.

**Table 1.** Patient Characteristics at Baseline

Patient Characteristics	Patients (*n* = 405)
Age (yr), mean (SD)	57.7 (13.4)
<50	95 (23%)
50–59	100 (25%)
60–69	145 (36%)
≥70	65 (16%)
Sex	
Female	328 (81%)
Male	77 (19%)
Race	
White	292 (72%)
Black	46 (11%)
Other	67 (17%)
Ethnicity	
Hispanic or Latino	62 (15%)
Other	343 (85%)
Cigarette smoking	
Never	272 (67%)
Former	112 (28%)
Current	21 (5%)
Diabetes	48 (12%)
Hypertension	114 (28%)
Sjögren's syndrome	38 (10%)
Thyroid dysfunction	80 (20%)
Rheumatoid arthritis	39 (10%)
Ever worn contact lenses	152 (38%)
OSDI total score (lower is better), mean (SD)	44.2 (14.2)
**Ocular Characteristics**	**Eyes (N = 794)**
Corneal staining score (lower is better), mean (SD)	3.5 (2.7)
Conjunctival staining score (lower is better), mean (SD)	2.9 (1.4)
Tearfilm tear break-up time (s) (higher is better), mean (SD)	3.1 (1.6)
Schirmer test (mm) (higher is better), mean (SD)	10.4 (7.1)
Keratography tear break-up time (higher is better), mean (SD)	8.3 (5.7)
Keratography bulbar redness score (lower is better), mean (SD)	1.15 (0.48)
Keratography tear meniscus height (higher is better), mean (SD)	0.38 (0.16)

The mean (SD) tear osmolarity was 303 (SD = 16) mOsm/L at baseline, 303 (SD = 18) mOsm/L at both 6 months and 12 months. Patients randomized to Omega-3 had a higher tear osmolarity than patients in the placebo group at baseline (304 vs. 301 mOsm/L; *P* = 0.03). However, the tear osmolarity did not differ between treatment groups at six months and 12 months (*P* ≥ 0.10; Table 2). The mean change from baseline at 12 months was a decrease of 0.6 mOsm/L in the omega-3 group and an increase of 3.4 mOsm/L in the placebo group yielding a difference of 4.0 mOsm/L (*P* = 0.03; Table 2). The percent of patients with abnormal tear osmolarity (defined as >308 mOsm/L in either eye or an inter-eye difference >8 mOsm/L) was 58% at baseline, 57% at six months, and 55% at 12 months; these rates did not differ between the two treatment groups (*P* ≥ 0.12; Table 2).

**Table 2.** Tear Osmolarity Measure at Baseline, Six, and 12 Months Over All and by Treatment Groups

Month	Statistic	All Combined	Omega-3	Placebo	Mean Difference (95% CI)	*P*
0	No. of eyes/no. of patients	794/405	520/265	274/140		
	Osmolarity (mOsm/L), mean (SD)	303 (16.2)	304 (17.0)	301 (14.4)	3.0 (0.3, 5.7)	0.03
	Patients with abnormal osmolarity^*^ (%)	236/405 (58%)	156 (59%)	80 (57%)	2.4% (−7.8%, 12.5%)	0.65
6	No. of eyes/no. of patients	676/346	446/229	230/117		
	Osmolarity (mOsm/L): Mean (SD)	303 (17.6)	304 (18.1)	301 (16.4)	2.7 (−0.5, 6.0)	0.10
	Patients with abnormal osmolarity^*^ (%)	196/346 (57%)	137 (60%)	59 (51%)	8.7% (−2.4%, 19.8%)	0.12
	Osmolarity change from baseline: mean (SD)	1.1 (19.1)	1.1 (19.5)	1.0 (18.3)	0.2 (−3.2, 3.5)	0.93
12	No. of eyes/no. of patients	695/357	462/237	233/120		
	Osmolarity (mOsm/L): Mean (SD)	303 (17.9)	303 (18.2)	303 (17.4)	−0.3 (−3.6, 3.0)	0.87
	Patients with abnormal osmolarity^*^ (%)	195/357 (55%)	127 (54%)	68 (57%)	−3.1% (−13.0%, 7.8%)	0.58
	Osmolarity change from baseline: mean (SD)	0.7 (20.3)	−0.6 (21.2)	3.4 (18.1)	−4.0 (−7.4, −0.5)	0.03

* Abnormal osmolarity defined as osmolarity >308 mOsm/L in either eye or an inter-eye difference >8 mOsm/L.

Comparing the tear osmolarity measured at summer (June, July, August) and winter (December, January, February) among 266 eyes that had osmolarity taken both in summer and winter, there was no statistically significant difference in osmolarity (303 vs. 301 mOsm/L; *P* = 0.09).

In the analysis of osmolarity data from baseline and six and 12 months combined, higher osmolarity was associated with older age (306 mOsm/L for age ≥70 years vs. 300 mOsm/L for age <50 years; *P* = 0.01) and presence of Sjögren's disease (311 vs. 302 mOsm/L; *P* < 0.0001). Osmolarity was not significantly associated with gender, race, ethnicity and systemic health status (*P* ≥ 0.13; Table 3). Similar results were found when data were analyzed separately at baseline and six and 12 months (See Supplementary Table S1).

**Table 3.** Association of Baseline Demographics With Osmolarity at Baseline, Six Months, and 12 Months Combined

Baseline Characteristic	Eye Visits *n* = 2165	Osmolarity (mOsm/L), Mean (SE)	*P*
Age (y)			0.01
<50	490	300.2 (1.1)	
50–59	539	303.6 (1.2)	
60–69	772	303.0 (0.9)	
≥70	364	305.8 (1.4)	
Sex			0.22
Female	1763	303.3 (0.6)	
Male	402	301.4 (1.5)	
Race			0.50
White	1585	302.7 (0.6)	
Black	233	305.4 (2.3)	
Other	347	302.9 (1.4)	
Ethnicity			0.13
Hispanic or Latino	314	301.2 (1.2)	
Other	1851	303.3 (0.6)	
Cigarette smoking			0.41
Never	1468	303.3 (0.7)	
Former	605	301.9 (1.0)	
Current	92	305.7 (3.7)	
Diabetes			0.67
No	1915	302.9 (0.6)	
Yes	250	303.6 (1.6)	
Hypertension			0.94
No	1566	303.0 (0.7)	
Yes	599	303.1 (1.1)	
Sjögren's syndrome			**<0.001**
No	1851	302.4 (0.6)	
Yes	192	310.6 (2.2)	
Thyroid dysfunction			0.92
No	1735	303.0 (0.6)	
Yes	430	302.9 (1.2)	
Rheumatoid arthritis			0.67
No	1974	302.9 (0.6)	
Yes	191	303.7 (1.8)	
Ever worn contact lenses—No	1331	303.6 (0.8)	0.15

The association between tear osmolarity and OSDI was evaluated by analyzing data from baseline and six and 12 months combined (Table 4) and separately (See Supplementary Table S2). In the combined analysis, the number of eyes in patient with osmolarity >308 mOsm/L was significantly associated with higher OSDI score (e.g., more severe dry eye symptoms), with mean OSDI score of 35, 37 and 39, respectively, for 0 eye, 1 eye, and 2 eyes, respectively (linear trend *P* = 0.04; Table 4). This association was significant at 12 months (*P* = 0.008) but not at baseline (*P* = 0.60) and six months (*P* = 0.40) (See Supplementary Table S2). The abnormal tear osmolarity (defined as >308 mOsm/L in either eye or an inter-eye difference >8 mOsm/L) and the osmolarity of worse eye (e.g., eye with higher osmolarity) were not associated with OSDI score in both combined analysis (Table 4) and separate analysis (See Supplementary Table S2). Change of osmolarity score from baseline at six and 12 months were not associated with change of OSDI score (*P* > 0.19; See Supplementary Table S3).

**Table 4.** Associations Between Person-Level Osmolarity and OSDI From Analysis of Combined Data From Baseline, Six Months, and 12 Months

Osmolarity	No. of Person Visits	OSDI Mean (SE)	*P*
No. of eyes with osmolarity >308 mOsm/L			
As categorical			0.13
0	645	35.3 (0.8)	
1	257	36.9 (1.3)	
2	155	39.1 (1.8)	
As continuous			
Pearson r	1057	0.07	**0.04**
Abnormal osmolarity^*^			
As categorical			0.79
No	481	36.1 (1.0)	
Yes	627	36.4 (0.9)	
Maximum tear osmolarity from two eyes of a patient (mOsms/L) (lower is better)			
As categorical			0.13
≤308	684	35.5 (0.8)	
>308 to ≤316	178	38.8 (1.6)	
>316	246	36.9 (1.5)	
As continuous			
Pearson *r*	1108	0.02	0.62

* Abnormal osmolarity defined at the person level as >308 mOsm/L in either eye or an intereye difference >8 mOsm/L.

The cross-sectional association of tear osmolarity with dry eye signs was evaluated by analyzing combined data from baseline and six and 12 months (Table 5). Higher osmolarity was significantly correlated with higher corneal staining score (Pearson correlation coefficient *r* = 0.17; *P* < 0.001), conjunctival staining score (*r* = 0.18; *P* < 0.001) and negatively associated with TBUT (*r* = −0.06, *P* = 0.03) and Schirmer test score (*r* = −0.07; *P* = 0.02). The significant associations of osmolarity with conjunctival staining score and corneal staining score remain when analyzed at each time point separately (all *r* ≥ 0.12; *P* < 0.004, See Supplementary Table S4). The change of osmolarity was not significantly correlated with change of dry eye signs (*P* ≥ 0.09, See Supplementary Table S5).

**Table 5.** Association Between Osmolarity With Dry Eye Signs and Symptoms From Analysis of Combined Data of Baseline, Six Months, And 12 Months

		OSDI		Conjunctival Staining Score		Corneal Staining Score		TBUT (sec)		Schirmer Test Score		
Osmolarity (mOsm/L)	No. of Eyes Visits	Mean (SE)	*P*	Mean (SE)	*P*	Mean (SE)	*P*	Mean (SE)	*P*	No. of eyes	Mean (SE)	*P*
Categorical									
≤308	1586	35.6 (0.8)		2.6 (0.1)		2.9 (0.1)		3.4 (0.1)		1576	10.8 (0.3)	
>308 to ≤316	263	38.7 (1.5)	0.08	2.8 (0.1)	**<0.001**	3.4 (0.2)	**<0.001**	3.4 (0.2)	**0.03**	260	10.3 (0.5)	**0.003**
>316	316	37.5 (1.7)		3.2 (0.1)		4.0 (0.3)		3.1 (0.1)		315	8.9 (0.5)	
As continuous										
Pearson *r*	2165	0.03	0.40	0.18	**<0.001**	0.17	**<0.001**	−0.06	**0.03**	2151	−0.07	**0.02**

In the analysis of person-level osmolarity abnormality (i.e., defined as osmolarity >308 mOsm/L in either eye or an intereye difference >8 mOsm/L) and maximum tear osmolarity from two eyes of a patient for their association with dry eye signs (Table 6), we found patients with abnormal osmolarity had significantly higher corneal staining score (*P* = 0.001) and conjunctival staining score (*P* = 0.007) and significantly lower TBUT (*P* = 0.04) and Schirmer test score (*P* = 0.01). Higher maximum osmolarity of two eyes of a patient was significantly correlated with higher corneal staining score (Pearson correlation coefficient *r* = 0.17; *P* < 0.001) and conjunctival staining score (*r* = 0.20; *P* < 0.001) and negatively correlated with TBUT (*r* = −0.07, *P* = 0.03) and Schirmer test score (*r* = −0.10; *P* = 0.009).

**Table 6.** Association Between Person-Level Osmolarity With Dry Eye Signs and Symptoms From Analysis of Combined Data of Baseline, Six Months, And 12 Months

		Conjunctival Staining Score		Corneal Staining Score		TBUT (Sec)		Schirmer Test Score	
Person-Level Osmolarity (mOsm/L)	No. of Person Visits	Mean (SE)	*P*	Mean (SE)	*P*	Mean (SE)	*P*	Mean (SE)	*P*
Abnormal osmolarity^*^									
No	481	2.5 (0.1)		2.8 (0.1)		3.6 (0.1)		11.1 (0.4)	
			**0.007**		**0.001**		**0.04**		**0.01**
Yes	627	2.8 (0.1)		3.3 (0.1)		3.3 (0.1)		10.1 (0.3)	
Maximum tear osmolarity from two eyes of a patient (mOsms/L) (lower is better)									
≤308	684	2.5 (0.1)		2.8 (0.1)		3.5 (0.1)		10.9 (0.4)	
>308 to ≤316	178	2.7 (0.1)	**<0.0001**	3.2 (0.2)	**0.001**	3.6 (0.2)	**0.06**	10.7 (0.6)	**0.02**
>316	246	3.1 (0.1)		3.8 (0.2)		3.1 (0.1)		9.2 (0.5)	
Pearson *r*	1108	0.20	**<0.0001**	0.17	**<0.0001**	−0.07	**0.03**	−0.10	**0.009**

* Abnormal osmolarity defined at the person level as >308 mOsm/L in either eye or an inter-eye difference >8 mOsm/L.

The association of tear osmolarity with keratography measures was analyzed at baseline and six and 12 months combined and separately (See Supplementary Table S6). In combined analyses, osmolarity was negatively correlated with keratography tear break-up time (*r* = −0.12; *P* < 0.0001), bulbar redness score (*r* = −0.09; *P* = 0.049), but was not correlated with tear meniscus height (*r* = 0.00; *P* = 0.93). Similar correlations between tear osmolarity and keratography break-up time was found at baseline (*r* = −0.12; *P* = 0.006), six months (*r* = −0.13; *P* = 0.01), and 12 months (*r* = −0.12; *P* = 0.02). However, change of tear osmolarity was not significantly correlated with change of keratography measures (*P* ≥ 0.09; See Supplementary Table S7).

## Discussion

There is no pathognomonic test universally recognized for definitive diagnosis of dry eye. DED is typically diagnosed by symptoms (sensation of dryness or foreign body sensation, excessive tearing, grittiness, eye fatigue, pruritis [itching], and fluctuating vision), and signs (fluorescein and lissamine green vital dye staining, tear film break-up time, and Schirmer testing). Abnormalities of tear osmolarity, i.e. hyperosmolarity has been reported to be key to the mechanism and pathogenesis of DED,^9^ therefore measurement of tear osmolarity was anticipated to be an appropriate metric for diagnosis and following treatment of DED, especially with the introduction of a point of care test in 2009 (TearLab Osmometer). A growing body of evidence, however, provides mixed results of the association of tear osmolarity with DED,^14^^,^^17^ leading to uncertainty of the usefulness of tear osmolarity measurements in clinical care of patients with DED. The DREAM study of 405 subjects with moderate to severe DED did not show correlation with symptoms as measured by OSDI but did show weak correlation with signs of DED.

The mean tear osmolarity was 303 at baseline and six and 12 months for the whole cohort. For analysis of treatment groups (omega-3 or placebo), the tear osmolarity was significantly different at baseline, but the same in both groups at six and 12 months. Tear osmolarity was increased with aging and increased in subjects diagnosed with Sjögren's syndrome. Tear osmolarity did not correlate with sex, race or systemic health status.

Although previous studies suggested tear osmolarity measurement was associated with presence or severity of dry eye disease and may offer a tool for the diagnosis or management of DED,^5^^,^^8^^,^^12^^,^^33^ various cutoff values were proposed to use for determining the abnormal osmolarity. Thus we evaluated the associations between tear osmolarity and OSDI in several ways including number of eyes with osmolarity >308 mOsm/L, presence of abnormal tear osmolarity (defined as >308 mOsm/L in either eye or an intereye difference >8 mOsm/L), and the level of osmolarity (≤308, >308 to ≤316, >316 mOsm/L) of the worse eye. These various analyses yield inconsistent results. We only found that patients with larger number of eyes with osmolarity >308 mOsm/L tended to have higher OSDI (linear trend *P* = 0.04). Future studies are needed to further investigate the associations between osmolarity and dry eye symptoms.

In this study, we found that patients with abnormal osmolarity had more severe dry eye signs, and magnitude of tear osmolarity was significantly correlated with dry eye signs as measured by corneal staining, conjunctival staining, TBUT and Schirmer's test. However, their correlation coefficients were all small ranging from −0.06 (for TBUT) to 0.20 (for conjunctival staining). Furthermore, changes in tear osmolarity over time did not correlate with changes in signs. Similar results were seen with keratography measurement of break up time and bulbar redness, but not with tear meniscus height. Changes in tear osmolarity were not significantly correlated with changes in keratography measurements. The lack of consistent correlations suggests only a weak correlation and one cannot deduce that hyperosmolarity is causative of ocular surface changes.

The strengths of the present study included the large data set, a multicenter trial, masked subject evaluation, and same subjects were examined over an extended period of one year. The weaknesses of this study are that we only studied moderate to severe DED in subjects that presented with both signs and symptoms, the study did not include normal subjects or those with mild DED. Although it is difficult to speculate, the results of this study might have changed if normal subjects or those with mild DED were included in this study. As such, it should be emphasized that there is a need for such studies. Tear osmolarity may not be an all or none measurement (one sample of the tear film is analyzed per eye) but may vary with location^34^ at which the tears are obtained (e.g., temporal vs. nasal environment of the inferior tear film meniscus sampled during testing, environmental conditions under which testing is performed, and may change rapidly over time). These characteristics are not easily measured with current device or current use limitations. There is the possibility that the number of osmolarity measurements at each visit is insufficient based on the findings where at least three consecutive measurements are required to provide clinically reliable tear osmolarity readings.^35^ Finally, we performed a large number of comparisons for evaluating the associations between osmolarity and DED symptoms and signs, and we did not correct for multiple comparisons, some of the significant findings can be due to chance.

In the DREAM study, tear osmolarity correlated weakly with ocular surface diseases, as defined by the signs measured in DED, and was associated with a known inflammatory disease, Sjögren's syndrome.^30^ In addition, increasing age is associated with increasing tear osmolarity. However, this minimally invasive tear osmolarity test did not correlate with DED symptoms as measured by the OSDI questionnaire, and further investigation is needed to find metrics that might provide an objective metric for symptoms. However, the DREAM study was designed to have dry eye evaluation based on practical real-world methods and the high consumable cost expense of the single use osmolarity test card to accomplish repeated measurements with the tear osmolarity instrument is impractical and economically precludes multiple osmolarity tests at one visit. Furthermore, refinement of tear osmolarity measurements such as regional measurements along the ocular surface and ability to do repeated measurements at one visit might provide additional information on its association with DED signs and symptoms.

## Supplementary Material

Supplement 1

Supplement 2
