# Supplementary material for: Association of Tear Osmolarity With Signs and Symptoms of Dry Eye Disease in the Dry Eye Assessment and Management (DREAM) Study
Source: Invest Ophthalmol Vis Sci. 2023 Jan 10;64(1):5. doi: 10.1167/iovs.64.1.5 (PMC9838582; doi:10.1167/iovs.64.1.5)
Supplement: Supplement 2 [file iovs-64-1-5_s002.pdf]

Table S1 (Online Supplement): Association of demographics with osmolarity at baseline, 6 months and 12 months

**Characteristic**		**Baseline**			**6 months**			**12 months**		
		**Eyes n=794**	**Osmolarity Mean (SE)**	**P**	**Eyes n=676**	**Osmolarity Mean (SE)**	**p**	**Eyes n=695**	**Osmolarity Mean (SE)**	**P**
Age (years)	<50	186	300.7 (1.4)	0.21	149	299.5 (1.4)	**0.04**	155	300.3 (1.4)	0.06
	50-59	197	302.4 (1.5)		173	305.3 (1.9)		169	303.3 (1.6)	
	60-69	283	302.8 (1.1)		242	303.6 (1.3)		247	302.6 (1.4)	
	≥70	128	305.5 (1.8)		112	304.2 (2.0)		124	307.4 (2.3)	
Sex	Female	642	302.7 (0.7)	0.97	551	303.6 (0.9)	0.32	570	303.8 (0.9)	0.054
	Male	152	302.6 (1.8)		125	301.5 (2.0)		125	299.7 (1.9)	
Race	White	572	302.4 (0.8)	0.18	497	302.4 (0.9)	0.15	516	303.2 (1.0)	0.21
	Black	90	306.5 (2.4)		71	303.3 (2.7)		72	306.1 (3.2)	
	Other	132	301.0 (1.8)		108	307.1 (2.3)		107	300.8 (1.5)	
Ethnicity	Hispanic or Latino	124	299.6 (1.7)	0.06	92	304.7 (2.2)	0.47	98	299.8 (1.3)	**0.01**
	Other	670	303.2 (0.8)		584	303.0 (0.9)		597	303.6 (0.9)	
Cigarette smoking	Never	536	302.3 (0.8)	0.26	457	303.5 (1.0)	0.86	475	304.1 (0.9)	0.23
	Former	217	302.1 (1.3)		194	302.8 (1.7)		194	300.8 (1.8)	
	Current	41	310.8 (5.2)		25	302.0 (3.2)		26	301.2 (4.6)	
Diabetes	No	700	302.3 (0.7)	0.14	597	303.0 (0.8)	0.51	618	303.5 (0.9)	0.08
	Yes	94	305.7 (2.2)		79	304.8 (2.6)		77	299.8 (1.9)	
Hypertension	No	571	302.1 (0.8)	0.22	490	302.9 (0.9)	0.56	505	304.0 (1.0)	0.07
	Yes	223	304.2 (1.5)		186	304.1 (1.7)		190	300.8 (1.5)	
Sjögren's syndrome	No	677	302.1 (0.7)	**<0.001**	575	302.7 (0.9)	**0.008**	599	302.5 (0.9)	0.0503
	Yes	74	310.8 (2.5)		61	311.0 (3.0)		57	309.8 (3.6)	
Thyroid dysfunction	No	640	302.8 (0.8)	0.59	540	303.4 (0.9)	0.69	555	302.9 (0.9)	0.56
	Yes	154	301.9 (1.4)		136	302.6 (1.7)		140	304.1 (2.0)	
Rheumatoid arthritis	No	718	302.6 (0.7)	0.95	618	302.8 (0.8)	0.19	638	303.3 (0.9)	0.30
	Yes	76	302.8 (2.3)		58	307.6 (3.6)		57	300.8 (2.2)	

**Characteristic**		**Baseline**			**6 months**			**12 months**		
		**Eyes n=794**	**Osmolarity Mean (SE)**	**P**	**Eyes n=676**	**Osmolarity Mean (SE)**	**p**	**Eyes n=695**	**Osmolarity Mean (SE)**	**P**
Ever worn contact lenses	No	493	303.2 (0.9)	0.35	409	304.1 (1.1)	0.15	429	303.6 (1.1)	0.39
	Yes	301	301.8 (1.1)		267	301.9 (1.2)		266	302.2 (1.2)	

Table S2 (online supplement): Associations between person-level osmolarity and OSDI

Osmolarity			Baseline			Month 6			Month 12		
			# persons	Mean (SE)	P	# persons	Mean (SE)	P	# persons	Mean (SE)	P
# of Eyes with osmolarity >308 mOsm/L	As categorical	0	232	44.1 (0.9)	0.84	199	31.1 (1.2)	0.59	214	29.8 (1.2)	**0.008**
		1	103	44.2 (1.4)		76	33.6 (2.5)		78	30.6 (2.2)	
		2	54	45.4 (2.1)		55	33.1 (3.1)		46	38.9 (2.7)	
	As continuous	Pearson r	389	0.03	0.60	330	0.05	0.40	338	0.14	**0.008**
Abnormal osmolarity*	As categorical	No	169	43.8 (1.1)	0.70	150	32.6 (1.5)	0.94	162	31.4 (1.5)	0.78
		Yes	236	44.4 (0.9)		196	32.4 (1.4)		195	30.8 (1.3)	
Maximum tear osmolarity from two eyes of a patient (mOsms/L) (lower is better)	As categorical	≤308	244	43.8 (0.9)	0.81	213	31.7 (1.2)	0.68	227	29.9 (1.2)	0.28
		>308-≤316	74	45.0 (1.7)		55	34.1 (2.9)		49	34.5 (3.0)	
		>316	87	44.3 (1.6)		78	33.3 (2.6)		81	32.3 (2.0)	
	As continuous	Pearson r	405	0.02	0.64	346	0.01	0.86	357	0.04	0.42

*Abnormal osmolarity defined at the person level as >308 mOsm/L in either eye or an inter-eye difference >8 mOsm/L.

Table S3 (online supplement): Associations between changes in person-level osmolarity and changes in OSDI

		Month 6			Month 12			All Combined		
	**Osmolarity (mOsm/L) change from baseline**	**# persons**	**OSDI change from Baseline Mean (SE)**	**P**	**# persons**	**OSDI change from Baseline Mean (SE)**	**P**	**# person visits**	**OSDI change from Baseline Mean (SE)**	**P**
As categorical	Decrease >5	118	-9.5 (1.7)	0.19	131	-13.2 (1.4)	0.99	249	-11.5 (1.3)	0.51
	Within ±5	105	-13.7 (1.5)		91	-13.3 (1.7)		196	-13.5 (1.3)	
	Increase >5	120	-12.1 (1.6)		129	-13.0 (1.5)		249	-12.6 (1.3)	
As continuous	Slope (SE)	343	-0.03 (0.05)	0.62	351	-0.02 (0.04)	0.66	694	-0.02 (0.04)	0.66
	Pearson r	343	-0.03	.	351	-0.02		694	-0.02	

Table S4 (online supplement): Association between osmolarity with dry eye signs and symptoms at baseline, months 6 and 12

	**Osmolarity (mOsm/L)**			**OSDI**		**Conjunctival staining score**		**Corneal staining score**		**TBUT (seconds)**		**Schirmer test score**		
**Month**			**# eyes**	**Mean (SE)**	**P**	**Mean (SE)**	**P**	**Mean (SE)**	**P**	**Mean (SE)**	**P**	**# eyes**	**Mean (SE)**	**P**
0	As categorical	≤308	579	44.0 (0.8)	0.72	2.9 (0.1)	**0.03**	3.2 (0.1)	**<0.001**	3.1 (0.1)	0.61	579	10.7 (0.4)	**0.02**
		>308 to≤316	101	45.3 (1.6)		3.0 (0.2)		4.2 (0.3)		3.0 (0.2)		101	10.2 (0.8)	
		>316	114	44.4 (1.7)		3.3 (0.2)		4.2 (0.3)		3.0 (0.2)		114	8.7 (0.6)	
	As continuous	Pearson r	794	0.01	0.79	0.12	**0.002**	0.15	**0.002**	-0.05	0.15	794	-0.10	**0.003**
6	As categorical	≤308	488	31.8 (1.1)	0.72	2.5 (0.1)	**<0.001**	2.7 (0.1)	**0.02**	3.7 (0.2)	**0.02**	483	11.0 (0.4)	**0.01**
		>308 to≤316	93	33.3 (2.5)		2.7 (0.2)		3.2 (0.3)		3.4 (0.3)		91	10.3 (0.8)	
		>316	95	33.7 (2.9)		3.3 (0.2)		3.9 (0.4)		3.0 (0.2)		94	8.6 (0.8)	
	As continuous	Pearson r	676	0.02	0.76	0.23	**<0.001**	0.20	**<0.001**	-0.09	**0.04**	668	-0.13	**0.002**
12	As categorical	≤308	519	30.0 (1.1)	**0.04**	2.4 (0.1)	**0.002**	2.7 (0.2)	**0.004**	3.6 (0.1)	0.18	514	10.8 (0.4)	0.32
		>308 to≤316	69	36.3 (2.8)		2.3 (0.2)		2.6 (0.3)		4.0 (0.3)		68	10.4 (0.8)	
		>316	107	33.6 (2.1)		3.1 (0.2)		4.0 (0.4)		3.3 (0.2)		107	9.5 (0.8)	
	As continuous	Pearson r	695	0.07	0.16	0.19	**<0.001**	0.18	**<0.001**	-0.03	0.35	689	0.01	0.90

Table S5 (online supplement): Association between change in osmolarity with change in dry eye signs and symptoms for months 6 and 12 separately and combined.

	**Change of osmolarity (mOsm/L) from baseline**			**Change of OSDI from baseline**		**Change of conjunctival staining score from baseline**		**Change of corneal staining score from baseline**		**Change of TBUT (seconds) from baseline**		**Change of Schirmer test score from baseline**		
**Month**			**# eyes**	**Mean (SE)**	**P**	**Mean (SE)**	**P**	**Mean (SE)**	**P**	**Mean (SE)**	**P**	**# eyes**	**Mean (SE)**	**P**
All	As categorical	Decrease >5	442	-11.5 (1.2)	0.33	-0.40 (0.07)	0.79	-0.53 (0.14)	0.52	0.32 (0.17)	0.45	440	0.37 (0.36)	0.42
		Within ±5	396	-13.3 (1.0)		-0.34 (0.08)		-0.51 (0.12)		0.55 (0.14)		391	-0.26 (0.43)	
		Increase >5	489	-12.5 (1.2)		-0.38 (0.07)		-0.67 (0.11)		0.55 (0.11)		484	0.13 (0.40)	
	As continuous	Pearson r	1327	-0.03	0.43	0.04	0.23	0.02	0.51	0.04	0.09	1315	-0.03	0.40
6	As categorical	Decrease >5	216	-9.8 (1.5)	0.17	-0.30 (0.09)	0.99	-0.41 (0.18)	0.46	0.47 (0.21)	0.75	214	0.72 (0.47)	0.33
		Within ±5	207	-12.9 (1.2)		-0.29 (0.10)		-0.63 (0.15)		0.58 (0.22)		205	-0.07 (0.53)	
		Increase >5	235	-12.7 (1.4)		-0.28 (0.09)		-0.67 (0.15)		0.40 (0.13)		232	-0.16 (0.47)	
	As continuous	Pearson r	658	-0.05	0.32	0.01	0.76	-0.02	0.58	-0.02	0.51	651	-0.06	0.12
12	As categorical	Decrease >5	226	-13.1 (1.3)	0.69	-0.50 (0.10)	0.73	-0.65 (0.16)	0.31	0.18 (0.18)	0.07	226	0.03 (0.48)	0.48
		Within ±5	189	-13.7 (1.3)		-0.39 (0.11)		-0.37 (0.17)		0.53 (0.16)		186	-0.46 (0.60)	
		Increase >5	254	-12.3 (1.3)		-0.46 (0.08)		-0.67 (0.15)		0.68 (0.13)		252	0.40 (0.50)	
	As continuous	Pearson r	669	-0.01	0.80	0.06	0.14	0.07	0.23	0.10	0.002	664	0.01	0.85

Table S6 (online supplement): Association between osmolarity with keratography measures for baseline, months 6 and 12 combined and separately

	Osmolarity (mOsm/L)		Keratograph break-up time			Bulbar redness score			Tear meniscus height		
**Month**			**# eyes**	**Mean (SE)**	**P**	**# eyes**	**Mean (SE)**	**P**	**# eyes**	**Mean (SE)**	**P**
Baseline, 6 and 12 months combined	As categorical	≤308	1092	8.4 (0.3)	**0.01**	1129	0.39 (0.01)	0.13	1064	1.16 (0.03)	0.89
		>308-≤316	161	7.3 (0.4)		167	0.36 (0.01)		148	1.14 (0.05)	
		>316	164	7.1 (0.4)		172	0.37 (0.03)		157	1.16 (0.06)	
	As continuous	Pearson r	1417	-0.12	**<0.001**	1468	-0.09	**0.049**	1369	0.00	0.93
Baseline	As categorical	≤308	394	8.7 (0.4)	**0.007**	398	0.39 (0.01)	0.37	373	1.15 (0.03)	0.75
		>308-≤316	77	6.8 (0.5)		78	0.36 (0.02)		69	1.17 (0.06)	
		>316	62	7.8 (0.7)		63	0.37 (0.03)		56	1.10 (0.07)	
	As continuous	Pearson r	533	-0.12	**0.006**	539	-0.09	0.07	498	-0.02	0.63
6 Months	As categorical	≤308	341	8.2 (0.3)	0.20	347	0.39 (0.01)	0.07	327	1.17 (0.04)	0.97
		>308-≤316	51	7.2 (0.8)		52	0.34 (0.02)		45	1.15 (0.07)	
		>316	53	7.0 (0.7)		55	0.38 (0.03)		47	1.16 (0.10)	
	As continuous	Pearson r	445	-0.13	**0.01**	454	-0.09	0.19	419	0.03	0.64
12 Months	As categorical	≤308	357	8.1 (0.4)	**0.02**	384	0.39 (0.01)	0.84	364	1.17 (0.03)	0.43
		>308-≤316	33	8.8 (0.9)		37	0.38 (0.04)		34	1.08 (0.08)	
		>316	49	6.2 (0.6)		54	0.37 (0.04)		54	1.21 (0.08)	
	As continuous	Pearson r	439	-0.12	**0.02**	475	-0.10	0.10	452	0.01	0.92

Table S7 (online supplement): Association between change of osmolarity with change of keratography measures for 6 months and 12 months combined and separately

	Change of osmolarity (mOsm/L) from baseline		Change of keratograph break-up time from baseline			Change of bulbar redness score from baseline			Change of tear meniscus height from baseline		
**Month**			**# eyes**	**Mean (SE)**	**P**	**# eyes**	**Mean (SE)**	**P**	**# eyes**	**Mean (SE)**	**P**
6 and 12 moths combined	As categorical	Decrease >5	290	-0.20 (0.45)	0.59	312	-0.002 (0.011)	0.68	266	-0.010 (0.027)	0.96
		Within ±5	269	-0.56 (0.43)		283	-0.001 (0.010)		255	-0.001 (0.024)	
		Increase >5	277	-0.87 (0.50)		293	-0.010 (0.010)		263	-0.001 (0.038)	
	As continuous	Pearson r	836	-0.02	0.56	888	-0.06	0.09	784	-0.03	0.62
6 moths	As categorical	Decrease >5	148	-0.34 (0.60)	0.89	152	-0.008 (0.011)	1.00	130	-0.002 (0.035)	1.00
		Within ±5	146	-0.71 (0.54)		149	-0.009 (0.011)		132	-0.002 (0.034)	
		Increase >5	132	-0.63 (0.64)		139	-0.009 (0.011)		118	0.001 (0.054)	
	As continuous	Pearson r	426	-0.01	0.86	440	-0.04	0.36	380	0.04	0.56
12 months	As categorical	Decrease >5	142	-0.05 (0.51)	0.41	160	0.004 (0.015)	0.45	136	-0.018 (0.036)	0.93
		Within ±5	123	-0.38 (0.59)		134	0.009 (0.013)		123	0.000 (0.034)	
		Increase >5	145	-1.09 (0.62)		154	-0.011 (0.013)		145	-0.002 (0.038)	
	As continuous	Pearson r	410	-0.04	0.47	448	-0.07	0.11	404	-0.09	0.18
